# Supplementary figures and images for: High expression in maize pollen correlates with genetic contributions to pollen fitness as well as with coordinated transcription from neighboring transposable elements
Source: PLoS Genet. 2020 Apr 1;16(4):e1008462. doi: 10.1371/journal.pgen.1008462 (PMC7112179; doi:10.1371/journal.pgen.1008462)

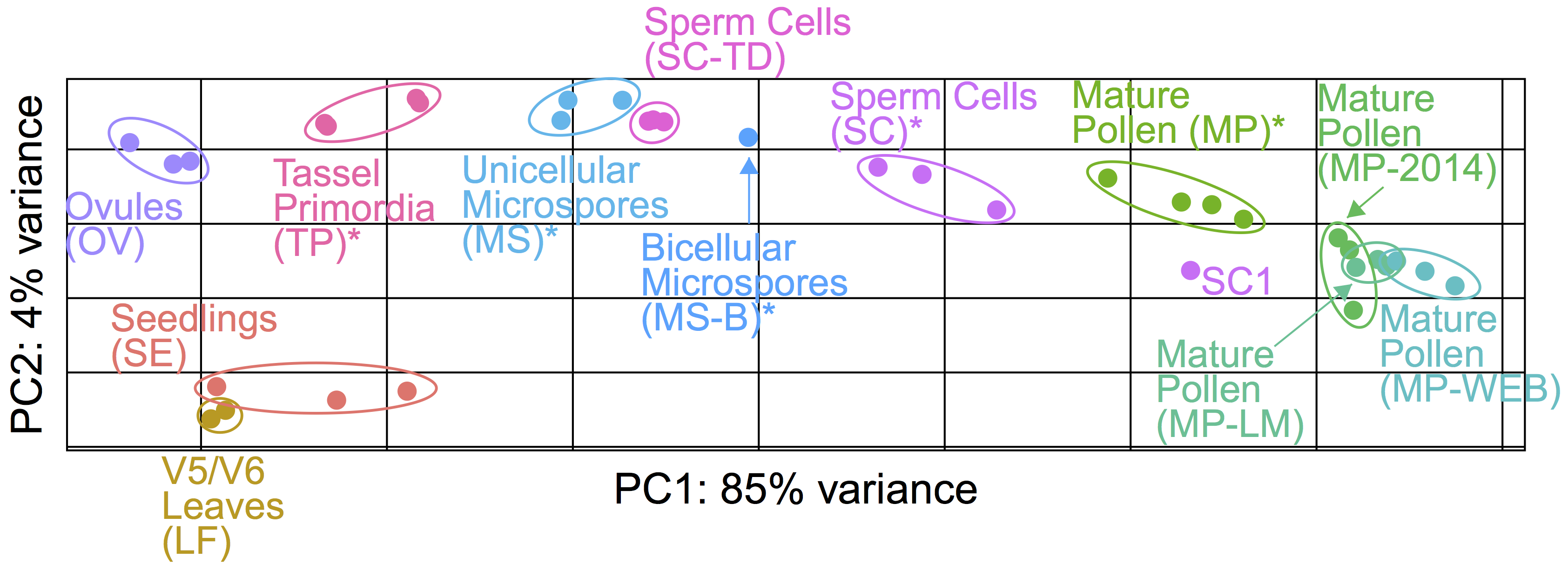

Supplement: S1 Fig — Two major components, on x- and y-axis, explain 89% of the variance in gene and TE expression levels. Asterisk (*) mark indicates the sample generated as part of this study, whereas other datasets are publicly available. For the sperm cells isolated in this study (SC), the TE expression of one biological replicate did not cluster with the other three (SC1), and therefore was removed from subsequent analyses of expression from TEs. MP-2014, SE, and OV are from [23]; MP-WEB is from [38]; LF is from [37]; MP-LM is from NCBI BioProject 306885 (2015); SC-TD is from [27]. (TIF) [file pgen.1008462.s001.tif]

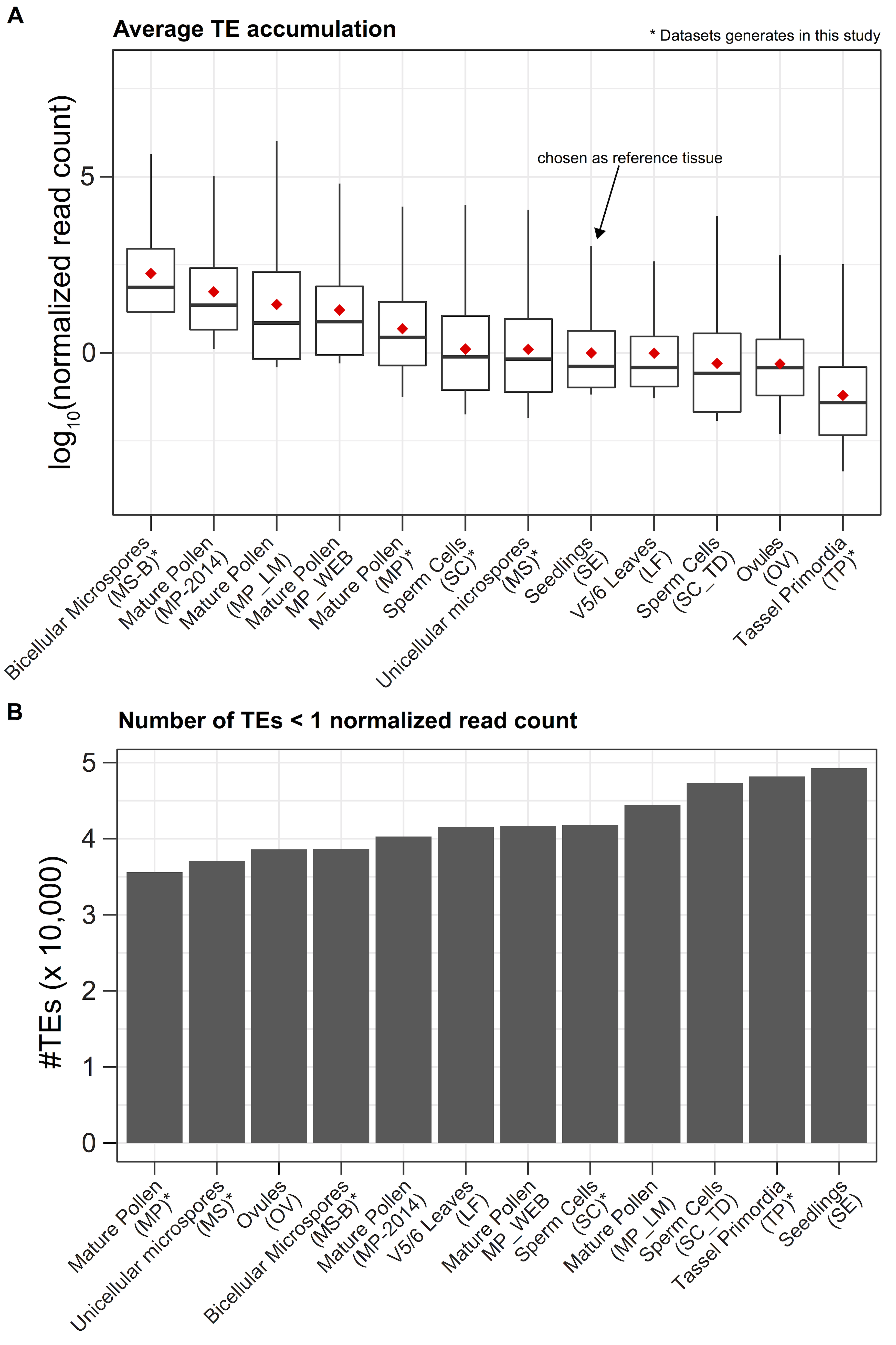

Supplement: S2 Fig — (A) Steady-state mRNA accumulation of all TEs in different tissues. Datasets generated in this study are marked with an asterisk. (B) The number of TEs with zero or near-zero expression levels in different tissues. Seedlings (SE) have the most TEs with low expression levels. (TIF) [file pgen.1008462.s002.tif]

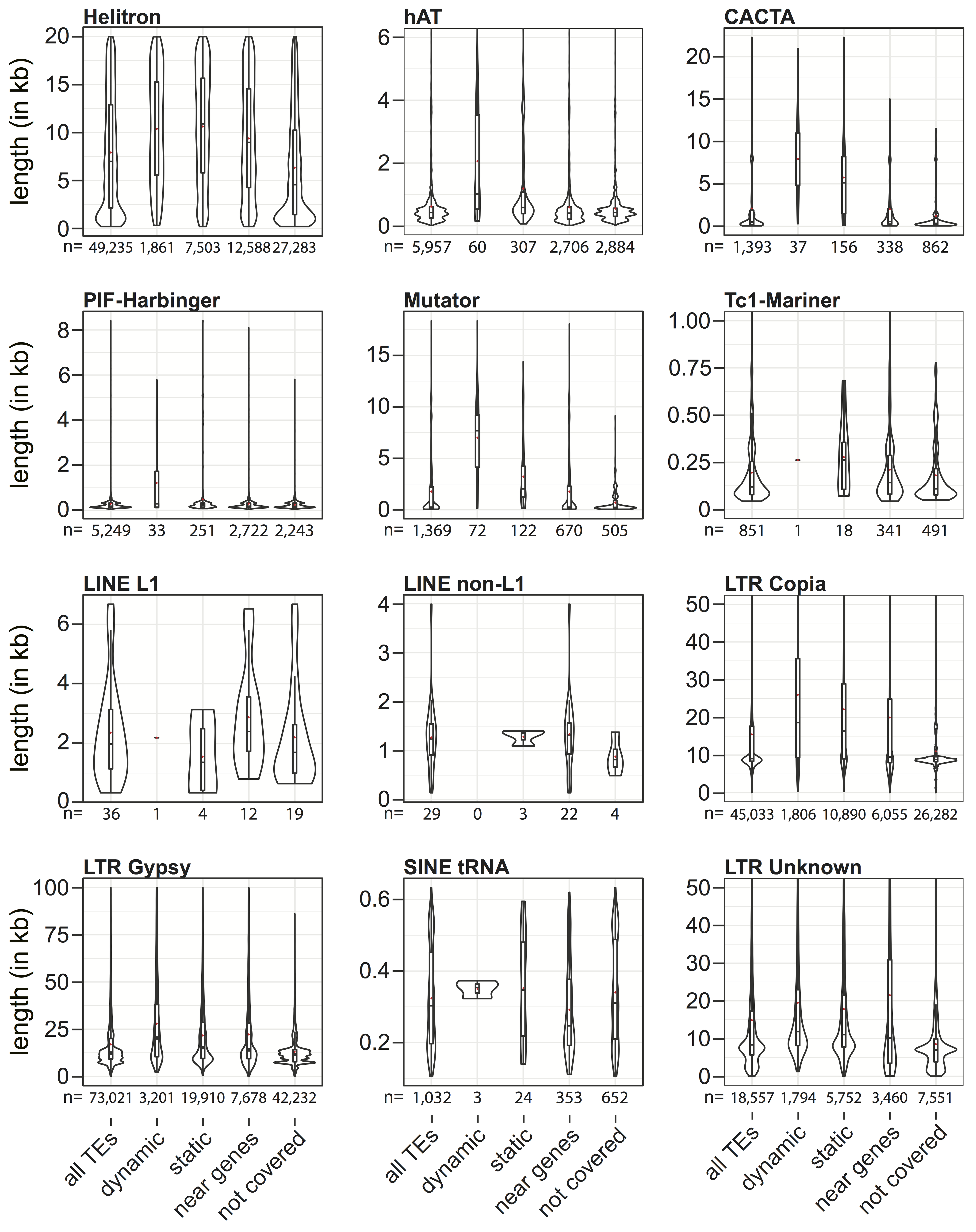

Supplement: S3 Fig — Length of TEs in the different TE categories from Fig 2A but further subcategorized by different superfamilies. The violin plots around the box show the kernel probability density of the data. The box represents lower and upper quartile, the line is the median, and the whiskers represent 10–90% range. Red asterisk denotes the mean. ‘n’ shows the number of TEs in each category for each superfamily. (TIF) [file pgen.1008462.s003.tif]

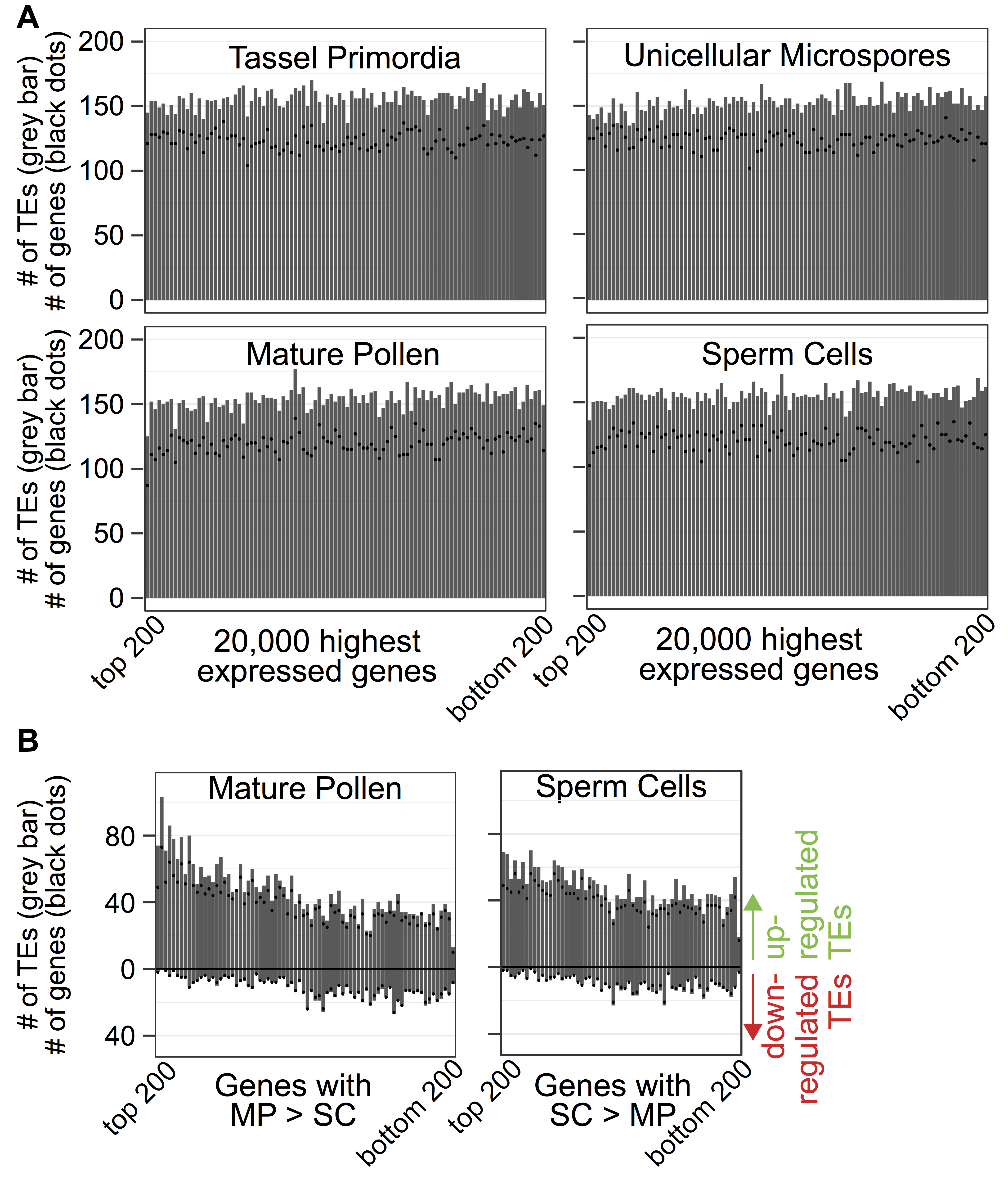

Supplement: S4 Fig — (A) For each tissue type, the top 20,000 expressed genes are distributed along the X-axis in bins of 200, with the highest expressed bin on the far left. The number of TEs near (<2kb) these genes is then counted on the Y-axis (shown in grey bar) and the number of genes with at least 1 TE within 2kb is displayed as black dots. (B) Genes filtered for either higher expression in pollen (MP) over sperm cells (SC) (left) or SC>MP (right) were used to determine if the association in Fig 4 is due to sample contamination between SC and MP. Once genes were filtered, the top expressed genes in that tissue were distributed along the X-axis in bins of 200 based on their expression values, with the highest expressed bin on the far left. The number of up- and down-regulated TEs near (<2kb) these genes is then counted on the Y-axis (shown in grey bar) and the number of genes with at least 1 TE within 2kb is displayed as black dots. (TIF) [file pgen.1008462.s004.tif]

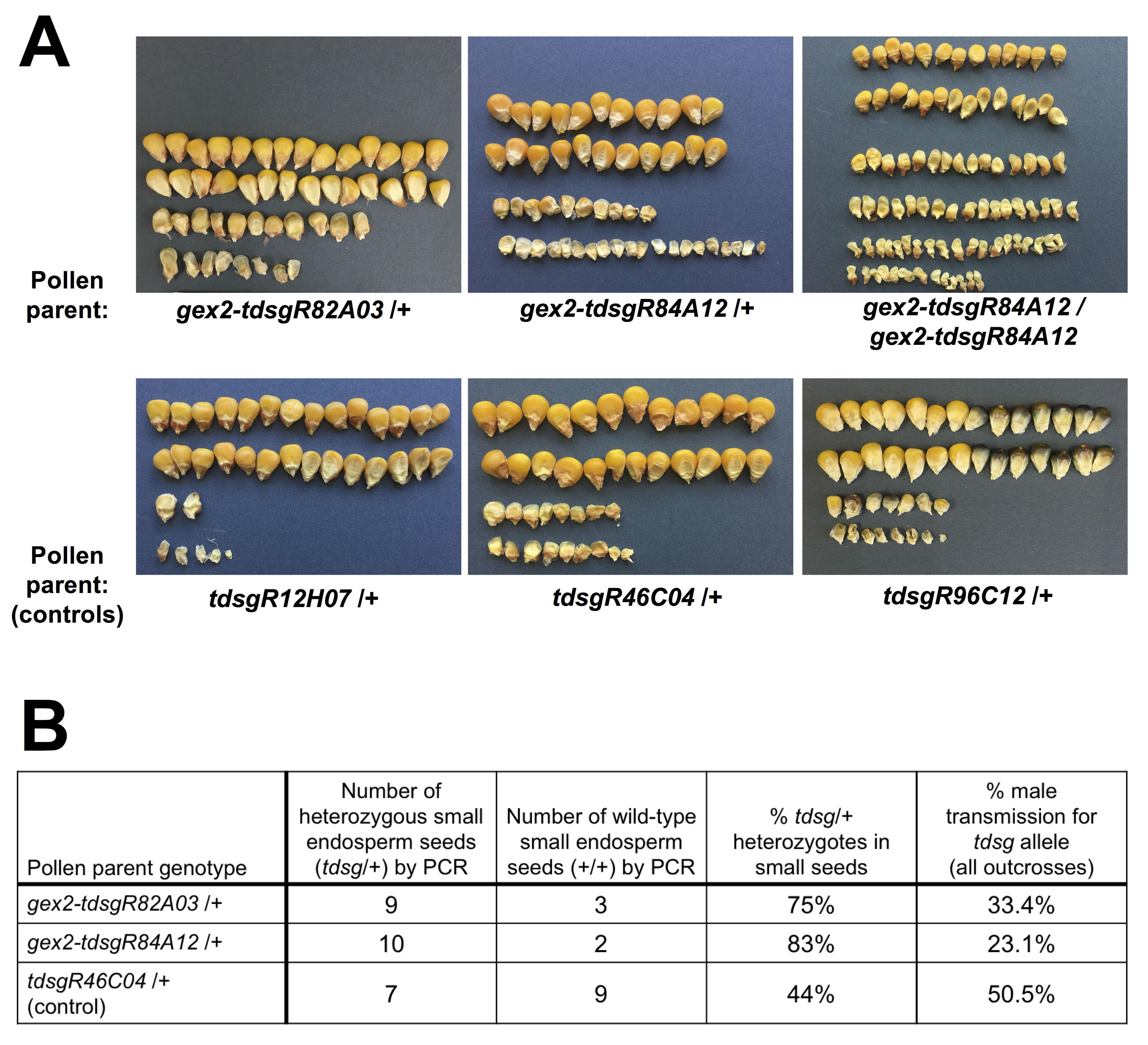

Supplement: S5 Fig — (A) Seeds were removed from ears, arranged according to size, and counted. Images of representative seed populations are shown, with the top two rows in each image showing representative fully developed seeds. Rows below the top two contain all of the smaller or aborted seed from that particular ear. (B) PCR genotyping of small endosperm seeds from two independent crosses for the two gex2 alleles show the majority of small seeds harbor the gex2::Ds-GFP allele, despite overall reduced transmission of the insertion alleles through the male. Small seeds from control tsdgR46C04 crosses segregate in a Mendelian fashion. (TIF) [file pgen.1008462.s005.tif]

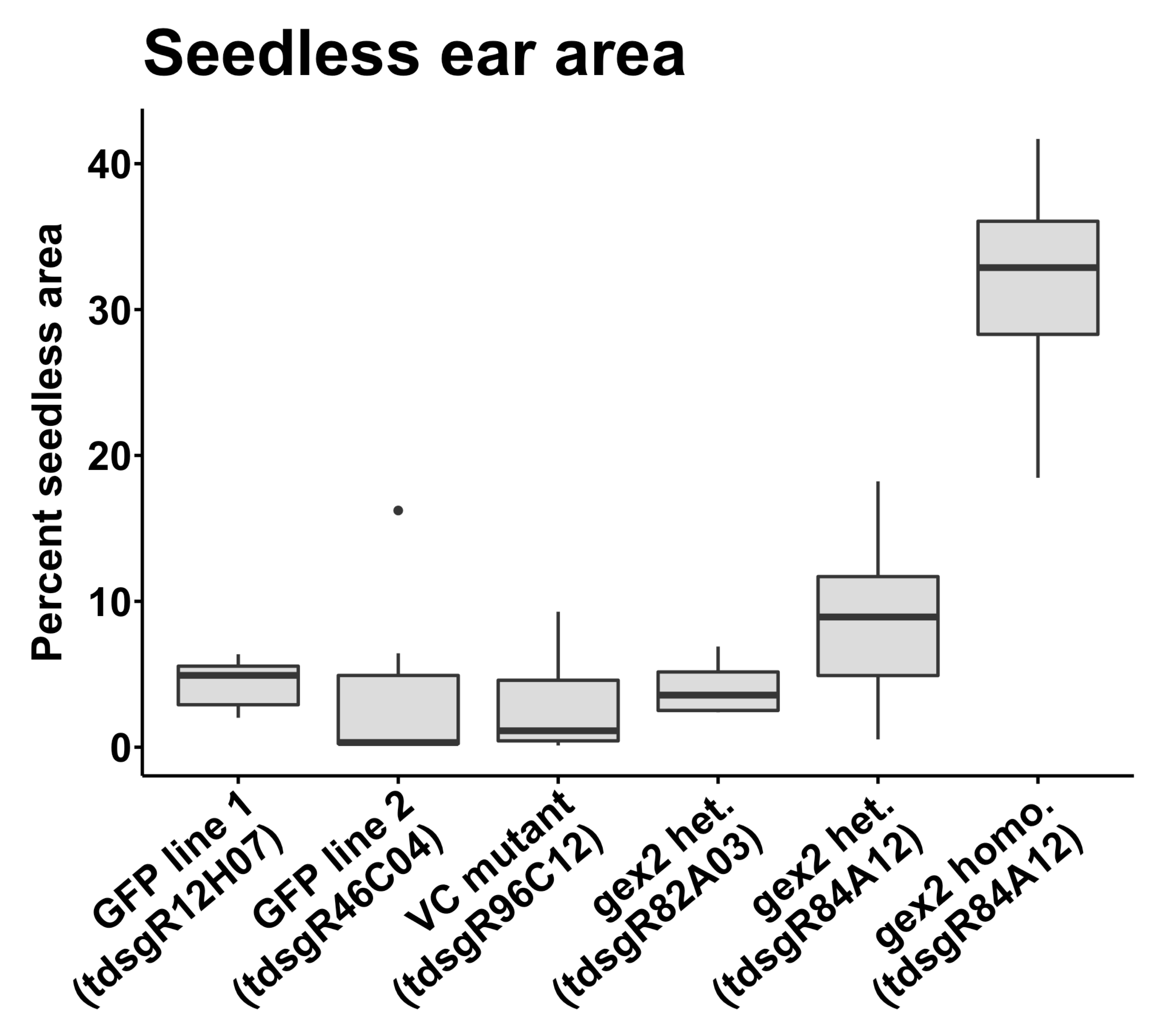

Supplement: S6 Fig — Seedless area was quantified from scanned ear images for gex2 Ds-GFP alleles and Ds-GFP controls. Pollen from heterozygous gex2 plants did not show significantly increased seedless area (gex2-tdsgR82A03 pairwise t-test p-values relative to GFP line 1, GFP line 2, and VC mutant 0.95, 0.96, and 0.74, respectively; gex2-tdsgR84A12 pairwise t-test p-values 0.19, 0.13, and 0.06, respectively), whereas pollen from homozygous gex2-tdsgR84A12 plants had significantly increased seedless area (pairwise t-test against Ds-GFP controls separately, all p-value < 0.0001). (TIF) [file pgen.1008462.s006.tif]
