# Supplementary material for: High expression in maize pollen correlates with genetic contributions to pollen fitness as well as with coordinated transcription from neighboring transposable elements
Source: PLoS Genet. 2020 Apr 1;16(4):e1008462. doi: 10.1371/journal.pgen.1008462 (PMC7112179; doi:10.1371/journal.pgen.1008462)
Supplement: S1 Methods — (PDF) [file pgen.1008462.s007.pdf]

S1 Methods. Tissue sample preparation, RNA extraction, and analysis of potential confounding variables in insertional mutagenesis lines.

Tissue sample preparations

Tassel primordia (TP)

Plants were grown in the greenhouse and cut at soil level when they reached V7 leaf stage. TP were 3-4 mm long at this stage, and were dissected out under a microscope from at least 4 (4-7) plants per sample, pooled, and immediately frozen in liquid nitrogen.

Microspores (MS)

Plants were grown in the field. On the day of isolation, tops of plants of the desired developmental stage were cut about 40 cm below the tassel. Cut stems were immediately placed in water. Tassels were unrolled from leaves and a total of 150 spikelets were removed from 3 different tassels (50 each). The tassels were placed in a 50 ml centrifuge tube containing 15 ml of chilled homogenizing buffer (HB) (0.5 M Mannitol, 1 mM EGTA, 20 mM HEPES pH 7.0). Samples of spikelets from each used plant were also set aside for anther measurement/developmental staging.

Spikelets in HB were homogenized on ice (Brinkmann Instruments, Polytron Kinematica PT10-35) using pulsed rather than continuous homogenization to insure material remained cold. The slurry was filtered through 100 μM and then 50 μM nylon mesh (BioDesign Inc. of New York) while kept cold. MS retained on top of 50 μM mesh were collected, resuspended in 5 ml of HB, and carefully centrifuged (65 g, 5 min, 4˚C). The MS pellet was gently resuspended in 5 ml of HB, loaded on top of 20%/35%/50% Percoll step gradient in a 50 ml centrifuge tube, and centrifuged (225 g, 10 min, 4˚C). MS fractions formed distinct bands at the border of 20% Percoll and HB (on top of the 20% Percoll layer) and at the border of 35% and 25% Percoll. The bands were individually collected using a syringe and 18G needle. The majority of MS in the 20% Percoll/HB fraction contained 1 nucleus, while most MS from the 35%/20% Percoll fraction contained 2 nuclei. MS were washed with 4 volumes of cold HB and centrifuged (100 g, 5 min, 4˚C). The MS pellet was transferred to a 1.5 ml Eppendorf centrifuge tube, HB was removed, and pelleted MS were used for RNA isolation.

The purity and developmental stage of isolated MS fractions was determined by light microscopy and by 4',6-diamino-2-phenylindole (DAPI) staining [1]. MS viability was assessed by fluorescein 3',6'-diacetate (FDA) treatment and fluorescent microscopy. To help determine pollen developmental stage of isolated MS, plants grown side-by-side with the set of experimental plants were monitored closely for time of anthesis. Using this method, we estimated that isolated MS were harvested approximately 10-14 days prior to anthesis.

Mature pollen (MP) and Sperm cells (SC)

Fresh MP was collected from plants in the greenhouse or field using standard maize pollen collection methods. MP from at least 5 (5-17) plants was pooled, cleaned of any anther debris, and weighed. MP was split into paired samples, with MP and SC RNA originating from the same pollen pool.

For each pair of MP and SC, 50 mg of pooled pollen was set aside for MP RNA isolation. The remaining pollen (at least 800 mg) was placed in a 15 ml Falcon tube and processed for SC isolation as follows: pollen was mixed with 520 mM Mannitol (100 mg/ml) at room temperature (RT), vortexed well to suspend pollen, then gently rocked for 10 min at RT. The tube with pollen was then moved to a rotator (Thermodyne slow speed Roto-Mix) at 60 rpm and 4˚C for an additional 20 min, then placed on ice.

The pollen suspension was filtered through 50 µM nylon mesh, then filtrate was collected into a plastic Petri dish on ice. The tubes and nylon mesh were rinsed with 2 ml of ice cold 520 mM Mannitol. The filtrate was then re-filtered through 22 µM nylon mesh and collected in a new plastic Petri dish imbedded on ice at an angle. The filtrate was carefully loaded onto a 7 or 8 ml ice-cold 10% Percoll cushion (10% Percoll in 15% Sucrose; 10 mM MOPS pH 7.5) and centrifuged (4500 g, 30 min, 4˚C). Following centrifugation, the SC layer was recovered from the Percoll/filtrate interface (densely opaque layer) using a 5 cc syringe with a blunt 18G needle. The collected SC suspension was gently added to 15-20 ml of ice-cold Sperm Isolation Buffer (SIB) (520 mM Mannitol,10 mM MOPS pH 7.4). Additional cold SIB was added so that a total of 4 volumes of cold SIB was mixed with sperm cells, followed by centrifugation (500 g, 15 min, 2˚C). Tubes were then placed on ice. The supernatant was removed using a serological pipette, then washed with 20 ml of ice-cold SIB. The pellet was resuspended by gently swirling tubes in an ice water bath, then centrifuged (500 g, 10 min, 2˚C). The SC concentrated in a definitive white to cream color pellet. SIB wash/supernatant was removed using a pipette. The SC pellet was resuspended quickly in ice-cold SIB to approximately 125 µl total volume while in an ice water bath. 100 µl of SC was transferred to a 1.5 ml cryotube, frozen in liquid nitrogen, and transferred to -80˚C. The remaining volume was kept on ice and used for SC concentration analysis using Vybrant Dyecycle Green (Invitrogen Cat # V35004) and cell viability assessment with FDA.

RNA isolation

Total RNA from TP, MS, and MP was extracted using the Trizol Reagent (Life Technologies) according to manufacturer's instructions with the following modifications for tissues with high polysaccharide content: 1) 20,000 MW PEG was added to the Trizol Reagent at 2% (20 mg/ml) concentration prior to homogenization by mortar and pestle; 2) During the precipitation step, a high salt precipitation solution (0.8 M Sodium citrate, 1.2 M Sodium chloride) was added to the aqueous phase, followed by Isopropyl alcohol as described in the manufacturer's instructions. A few samples (TP3, MS2, MS3) were further purified using an RNeasy MinElute Cleanup Kit (Qiagen Cat # 74204) according to manufacturer's instructions, with the modification of adding 20,000 MW PEG at 3.3% (33 mg/1 ml) to the RLT buffer..

Total RNA from SC was extracted by phenol-chloroform extraction using RE Buffer (0.1 M Tris pH 8.0, 5 mM EDTA pH 8.0, 0.1 M NaCl, 0.5% SDS, 10 µl fresh 2-mercaptoethanol per 1 ml of RE buffer) [2]. RNA (mRNA) was isolated using streptavidin magnetic beads (New England Biolabs, # S1420S) and a biotin-linked poly-T primer [3].

Analysis of potential confounding variables in insertional mutagenesis lines

One potential confounding variable in the insertional mutagenesis dataset (see "Large-scale insertional mutagenesis supports a relationship between transcript level and fitness contribution for vegetative cell-expressed genes") was the presence of the *wx1-m7::Ac* allele in 11 out of 56 (19.6%) of the lines tested (S7 Table). This *Ac* was used for the generation of the original *Ds-GFP* transpositions [4], and the element remained in 21 out of 166 (12.7%) of male parents in the study, via PCR. Because *Ac* transposition is a rare event [5,6] and germinal *Ds* transposition rates are only a few percent [7], we expected the presence of *Ac* to have little or no detectable effect on transmission rates. In the 8 families where *wx1-m7::Ac* was present in some parents and absent in others, we saw a non-significant difference in transmission rates between progeny of those plants that contained *wx1-m7::Ac* (47.1% insertion allele transmission) and of those that did not (49.3% insertion allele transmission) (quasi likelihood test on generalized linear model, p-value = 0.84). Importantly, aside from *gex2*, none of the lines associated with significant non-Mendelian inheritance harbored *wx1-m7::Ac*. For *gex2*, *wx1-m7::Ac* was present in all parents carrying one *gex2* allele, but completely absent in crosses with second *gex2* allele, yet both alleles showed similarly reduced transmission (Fig 6C). For these reasons, we argue that the presence of *wx1-m7::Ac* does not significantly impact *Ds-GFP* and/or allele transmission rates presented in the dataset. We also considered the possibility that reduced frequency of the GFP seed phenotype in some lines was due to a fraction of progeny transmitting epigenetically silenced GFP transgenes through the male, rather than aberrant transmission of the insertional allele itself. However, PCR genotyping of progeny from male crosses in five of the aberrant lines (with male transmission rates of 23%, 30%, 33%, 44% and 44%) all showed perfect co-segregation of the GFP or non-fluorescent endosperm phenotype with the respective, expected genotype (S9 Table), arguing against transgene silencing as an explanation. For 46 of the 52 genes tested, only single *Ds-GFP* alleles were available, leaving open the possibility that any defects found in these were caused by linked mutations, rather than insertion into the target gene. The female segregation ratios we observed were consistent with single *Ds-GFP* insertions in all plants used for assessing male transmission rates, arguing against widespread off-target insertional mutations in these lines. In addition, for the four genes tested with two independent insertions each, consistent transmission rates were seen for both alleles (Fig 6C & D, circled pairs – three with no significant defect, one with a defect), supporting the contention that the experimental design is robust to variation in insertion sites within each gene, and that off-target effects in these lines are uncommon.

References

1. Heuer S, Lörz H, Dresselhaus T. The MADS box gene ZmMADS2 is specifically expressed in maize pollen and during maize pollen tube growth. Sex Plant Reprod. 2000;13: 21–27.
2. Box MS, Coustham V, Dean C, Mylne JS. Protocol: A simple phenol-based method for 96-well extraction of high quality RNA from Arabidopsis. Plant Methods. 2011;7: 7.
3. Townsley BT, Covington MF, Ichihashi Y, Zumstein K, Sinha NR. BrAD-seq: Breath

Adapter Directional sequencing: a streamlined, ultra-simple and fast library preparation protocol for strand specific mRNA library construction. Front Plant Sci. 2015;6: 366.

4. Li Y, Segal G, Wang Q, Dooner HK. Gene Tagging with Engineered Ds Elements in Maize. In: Peterson T, editor. Plant Transposable Elements: Methods and Protocols. Totowa, NJ: Humana Press; 2013. pp. 83–99.
5. McClintock B. Chromosome organization and genic expression. Cold Spring Harb Symp Quant Biol. 1951;16: 13–47.
6. Levy AA, Walbot V. Regulation of the timing of transposable element excision during maize development. Science. 1990;248: 1534–1537.
7. Vollbrecht E, Duvick J, Schares JP, Ahern KR, Deewatthanawong P, Xu L, et al. Genome-wide distribution of transposed Dissociation elements in maize. Plant Cell. 2010;22: 1667–1685.
