# Supplementary material for: High expression in maize pollen correlates with genetic contributions to pollen fitness as well as with coordinated transcription from neighboring transposable elements
Source: PLoS Genet. 2020 Apr 1;16(4):e1008462. doi: 10.1371/journal.pgen.1008462 (PMC7112179; doi:10.1371/journal.pgen.1008462)
Supplement: Attachment — Submitted filename: Response to Reviewers_Revision1.docx [file pgen.1008462.s019.docx]

**RESPONSE TO REVIEWERS**

**PLOS Genetics (PGENETICS-D-19-01653) - [EMID:2bcfe2130d1eca87]**

Dear Editors and Reviewers,

Thank you for your time and effort on our submitted manuscript, and for allowing us to submit a Revision. We have extensively revised the manuscript, based on Reviewer and Editor comments, including restructuring the Discussion, tightening the narrative, cutting the length, adding analyses, and generating new figures. We believe that the manuscript is markedly improved over the first submission, and thank you for the feedback, which allowed us to focus on aspects that needed improvement. We included both a ‘clean’ version of the manuscript, as well as a version with the changed sections identified by green highlighting. In our Response to Reviewers, the original reviewer comments are reproduced and highlighted in yellow, and our response to each point follows.

A side, process note: As mentioned below, the addition of requested data to Tables 1 and 2 significantly increased the size of these tables, such that they could not be fit into the manuscript (as a Word document) without reducing the font size used in the Tables - this approach was taken in this submission. We will happily provide versions of these Tables as separate files in an alternate format (e.g., .pdf, .xlsx) if requested.

We remain enthusiastic about this work, and very positive about the possibility of seeing it published in *PLOS Genetics.*

Thank you for your consideration,

John Fowler

*************************************

**Comments to the Authors:**

**Reviewer #1:**

I think the title is problematic, because of the word “and”. The paper has two parts: in the first they demonstrate TE expression in male development similar to what was shown for Arabidopsis, but find key differences in timing and location. No connection between TE part of the paper and the transmission defects part of the paper; could be a coincidence, nothing more.

We have reworded the title and extensively revised the manuscript structure and text, aiming to remove any suggestion that we have identified a set of pollen genes that all have the following characteristics: highly expressed, coordinately regulated with neighboring TEs, and contributing to pollen fitness. Based on comments from the Reviewers #1 and #2, our language and the structure of the manuscript was unclear on this point. Based on comments from the Editor, our revision is revised throughout to highlight that there are two threads connecting the different parts of the manuscript (e.g., lines 171-181; lines 315-317). The first (implicit) thread is the RNA-seq dataset itself, described in the first part and used throughout to enable the subsequent parts of our study (extensive TE expression analysis; mutational interrogation of gene function; identification of the gex2 phenotype). The second thread is the exploration of aspects of the population of genes that are ‘highly expressed’ in pollen, based on the transcriptome dataset. Our data point toward two interesting aspects of this population of genes in mature pollen: 1) a tendency to be adjacent to transcriptionally-active TEs; and 2) a tendency to encode genes required for Mendelian segregation (i.e., contributing to pollen fitness). The revision works to clarify that, although these are both aspects of ‘highly expressed pollen genes’, these two tendencies are not correlated to any major extent (this is addressed explicitly in the next-to-last paragraph in the Discussion).

Much of work on the GEX2 mutant is duplicative of what was already published in Arabidopsis.

We have added sentences in multiple locations (lines 53-55; 418-423; 438-439; 452; 472-479) to more extensively acknowledge the previous Arabidopsis results with GEX2, and to highlight that our data support the idea that a functional role for GEX2 in double fertilization is conserved across flowering plants.

Transmission defects for vegetative-expressed genes are modest, except for one –surprised they found anything, as many pollen proteins are members of multi-gene families (all expressed in pollen). So gene redundancy might be expected to cover for any defect in another. They should provide info. about possible gene redundancy to explain lack of/presence of phenotypes for the genes they chose to test. I am not convinced by their idea that highly expressed genes are likely to be more important for function.

Our general goal in this part of the project was to validate the biological significance of the transcriptome analysis, i.e., test the hypothesis that transcript level provides meaningful information relevant to biological function (in this case, measured by a quantitative mutant phenotype). We note that the hypothesis proposed (higher transcript level is more likely to correlate with biological function) does not rule out or obviate the contribution of other factors to function/phenotype in a complex biological system - e.g., genetic redundancy (as suggested by the Reviewer), translational efficiency, protein type (e.g. enzyme vs. transcription factor). However, given the widespread availability of transcriptome data, it seemed a useful initial hypothesis to test formally, taking advantage of the quantitative framework provided by our measurements for transcript level, and analyzing their relationship to measurements of transmission frequency.

We stress that, after choosing a ‘Top 20% by FPKM’ cutoff for potential genes to study, the genes tested were chosen randomly from this larger set to provide as unbiased a sample as possible. The statistical analysis depicted in Fig. 6 does show correlation between transcript level and the phenotypic measures, but it is limited (R^2^ < 0.2). Given the relatively small sample size, and the fact that there is potential bias due to the limited availability of genes for assessment via mutation (see response to Reviewer #3 below), we respect Reviewer #1’s perspective of “not convinced”, and have rewritten several relevant sentences in the manuscript to qualify our results (lines 388-393 regarding the correlation and R^2^ ; lines 505-508 in the Discussion).

To address Reviewer #1’s hypothesis, we have now assessed another potential factor influencing phenotype, genetic redundancy. Tables 1 and 2 (encompassing all genes interrogated by *Ds-GFP* insertion) now provide the character ‘Gene Type’, i.e., whether each gene is a singleton or a syntelog in a retained pair of syntenous genes derived from the ancestral maize genome duplication. As presented in the Discussion (lines 509-513), this analysis does not show a significant correlation between the ‘singleton’ character and non-Mendelian segregation, although the trend seen (6 of 7 non-Mendelian genes are singletons, whereas 14 of 25 Mendelian genes are singletons) certainly suggests that redundancy is likely to be a factor, and worth additional experimentation in a followup project.

Finally, we note that a study we had missed in our earlier submission (Liao and Weng 2015), a meta-analysis in mouse, found a somewhat similar linkage between transcriptome data and mutant phenotype; this reference is now worked into the Discussion (line 532-534).

Paper is not user-friendly – e.g., if a reader is interested in knowing what genes were tested and which ones did not show transmission defects, the supplemental tables don’t help, long gene identifier numbers, nothing else. A table of annotations for all tested genes should be in the body of the paper.

We have added [Table 2](https://docs.google.com/spreadsheets/d/1bPaMmP2VAlfujr5tM-zrSbJVXsd4AjsMnasJz_3Dy34/edit?usp=sharing), which, alongside Table 1, provides the requested annotations for the entire set of genes tested for transmission defects in our study. Due to the additional data now included in these Tables, we needed to dramatically decrease the font size to allow inclusion of the Tables in the manuscript (Word) document.

Line 2: strange to say that the male gametophyte is essential for subsequent initiation of seed development – with such an argument, it must be important for every subsequent step in the life cycle. Just say pollen tube growth and sperm delivery.

We removed "and subsequent initiation of seed development" from line 36.

Line 45: insert the word the in front of the word pollen

Done (line 47).

Lines 50-52: a role for GEX2 already demonstrated in Arabidopsis (reference 47); should be mentioned here. Similarly, in line 69- role for GEX2 in fertilization already established – if the authors insist on this phrasing, they need to say maize gex2 throughout. Similarly in lines 138-140.

We have altered the text throughout, using the prefix ‘Zm’ where necessary, to clarify when the gene is from Arabidopsis or maize, following the MaizeGDB nomenclature recommendations: “the use of the prefix *Zm* in front of the gene's name will only be used when comparing maize genes with related genes from other species (e.g., *Zm myb1*).” (e.g., line 52; line 407; lines 418-420) In addition, we note that the maize and Arabidopsis nomenclature conventions also distinguish the genes, as maize uses all lower case letters (*gex2*), whereas Arabidopsis uses all upper case (*GEX2*).

As requested, we have added a sentence to the abstract to reference the Arabidopsis GEX2 results (lines 54-55).

Line 61; what does “this process” refer to? Pollen tube growth?

Replaced "this process" with "sperm cell delivery" (line 63)

Line 240 – the word somatic is not needed

The word “somatic “ was deleted

Line 272, vice, not vise

This requested change has been made (line 290)

Line 283-285, they should also admit “or neither”, i.e. expression levels of TE and genes could be a coincidence, neither affecting the other.

We have adjusted this sentence in the manuscript text. We note that the specific association between nearby gene/TE expression level that is apparent in the lower panels of Fig 4 argues against coincidence, as does the newly included statistical analysis (lines 293-300, revised Figure 4 & legend). However, based on Reviewer #1’s comment, we have added that this could be due to a higher order global regulatory mechanism (line 310).

Line 289, there is no such thing as high or low tissue-specificity, either something is specific or it is not. Otherwise use the word enriched.

We revised this line to substitute ‘enriched’ for ‘specific’. This sentence was in the paragraph moved to earlier in the ms (now the final paragraph in the first section of Results) (line 172).

Line 307 – competitive delivery of gametes is unusual phrasing, the competition is at the level of pollen tube growth.

Our purpose in the phrasing we chose was to encompass all aspects of the competitive process leading up to delivery of the sperm cells to the embryo sac. It is true that competition during pollen tube growth likely plays the major role; however, differences in the frequency of germination, as well as the rate of pollen hydration and of pollen tube germination, may also lead to differences in the final outcome, without affecting pollen tube growth *per se*. We have chosen to maintain the phrasing as in the original submission (line 322).

Line 356-362. I don’t understand the math – 6 of 7 and then 6 of 12.

These were referring to two different groups of genes: 1. six with mild defects our of seven total with significant defects; and 2. six with significant defects out of twelve total expressed with log2(FPKM)>8. We have shortened and clarified this section in the text (lines 383-88).

Line 394, cite ref. 46 after EST sequencing, and the reference to the name GEX2 (ref 50), is missing. Neither ref should be at the end of the sentence, as “no mutant has been described” is not mentioned in either ref. 46 or ref 50. The statements on lines 495-497 are better and accurate.

Line 400 “has been described” is passive voice – Ref. 47 shows it.

Line 401 – of course it encoded a protein similar to Arabidopsis Gex2, the way Gex2 was identified was by using maize ESTs (ref. 46) to find the Arabidopsis homolog (ref 50).

This paragraph has been extensively revised to 1) address referencing concerns; 2) alter the passive voice; and 3) generate a more concise description of the maize protein and its relationship to the Arabidopsis ortholog (lines 418-423).

Lines 405-441 essentially are showing in maize what was already shown (ref 47) in Arabidopsis. Probably does not merit this much space.

These two paragraphs were extensively revised and shortened, as requested by the reviewer (lines 438-453). We maintained aspects of the description that 1) highlight experimental data relevant to GEX2 function that were not presented by the Mori et al paper (e.g., PCR genotyping of aborted seeds to assess whether these are induced by fertilization with gex2 mutant sperm); 2) feature elements particular to our approach with imaging maize ears (e.g., quantifying seedless area on gex2-pollinated ears); and 3) enable a reader unfamiliar with previous work on GEX2 to understand the phenotypes found.

Line 507-508 Why mention “we cannot rule out….”

Removed sentence.

Lines 512-520 – why discuss these other mutants? They don’t bear on the work in this paper.

The gex2 discussion section has been dramatically shortened to a single paragraph, including removal of the paragraph comparing the gex2 phenotype to other maize mutants with similar effects (lines 461-479).

Lines 555 mild to moderate is an overstatement here, 5 were mild, only one was moderate.

We have kept our original phrasing, as it described the range of effects detected, and have added a clause to indicate that the majority of the defects are mild (lines 381-383; lines 502-505).

Line 560 – delete extrapolation comment, it is pointless from such a small sample.

Done.

Discussion is much too long, could be cut by 40%.

We have significantly revised the Discussion, cutting throughout, including the sections specifically identified by Reviewer 1. However, we also needed to add language to address points of concern raised by all three Reviewers, and in the end, did not meet the 40% target given by Reviewer 1. We feel that additional cuts would remove important points that help establish the broader relevance of this work. We ask that, if Reviewers still find the Discussion too lengthy, to provide additional specific guidance regarding sections that they believe are less useful for this purpose.

*************************************

**Reviewer #2:**

In this manuscript by Warman et al., the authors performed RNA-seq at different stages of pollen development to analyze the expression patterns of both genes and transposable elements and then screened mutants in highly expressed genes for transmission defects that imply functional importance of these genes. The highlight of this manuscript was the use of GFP-marked insertion mutants followed by clever imaging to identify genes that reduce pollen fitness. The authors then characterized the aborted seed phenotype seen in paternally inherited gex2 mutants, showing that this gene is involved in double fertilization.

I do have some concerns about this manuscript, specifically with the analysis of the transcriptome data and the integration of transcriptome data with the analysis of transmission defects. For example, the title is misleading. The authors show that several highly expressed maize pollen genes contribute to pollen fitness, but as far as I could tell, only one of these functionally tested genes is near a TE with coordinated expression.

Please see the altered title, and the related first response to Reviewer 1 above.

Furthermore, the result that highly expressed genes are enriched for up-regulated TEs nearby is interesting but is not well supported by the data presented. What I could not determine from the figures is how many genes have at least one up-regulated TE within 2 kb or how TEs that overlap genes are treated. In Figure 4A, the y-axis shows the number of TEs within 2 kb of bins of 200 genes, but since a single gene may have multiple up-regulated TEs within that vicinity, it is not clear how many genes are associated with up-regulated TEs.

We have performed the requested analysis and integrated these data into Figures 4 and S4. These figures now show the number of genes with at least one TE within 2kb that is either up or down regulated. This pattern of number of genes closely mirrors the number of TEs, demonstrating that the association discovered is not due to one gene being located near many differentially expressed TEs. This also further supports our conclusion that there is co-regulation between TEs and neighboring genes specifically in the pollen grain and sperm cells (lines 302-307, Figure 4 legend).

With respect to overlap, the authors acknowledge earlier in the manuscript that TEs within 2 kb of a gene introduce complications due to overlaps and the potential for read-through transcription, so it is not clear why this major conclusion of the paper is not more thoroughly assessed to rule out this possibility.

In the first two sections of the TE analysis, we removed “near gene” TEs to ensure that we are investigating TE-initiated TE expression and not transcriptional read-through from protein-coding genes. After assessing this set of TE’s, we then moved to assess the separate set of near-gene TEs to investigate co-regulation in this third section of analysis - the difference between the two sets of TEs is now more clearly emphasized in the ms (lines 199-202; 290-291). In this manuscript, we make no claim as to whether the TE/gene co-regulation is transcription from both the gene and TE, or from one transcript that spans both (this point has been added to the discussion section – lines 589-590). It is not possible from our short-read dataset to determine the location of transcriptional start sites in regions with multiple overlapping transcripts, i.e., to determine what is read-through. This would take CAGE or long-read transcriptome sequencing.

The conclusion that TE transcription in maize is largely static across development is surprising and contradicts prior studies in maize using EST or RNA-seq data, but this difference is not addressed in the manuscript.

We find that only ~21% of annotated maize TEs are static. Furthermore, these TEs are also all in the subset that are >2 kb from genes, have measurable expression levels, and have polymorphisms that allow mapping reads specifically to those individual TEs. We are therefore not claiming that TE transcription is largely static across development. The majority of maize TEs (55%) are categorized as ‘not covered’ in this study: TEs with very low levels of expression or highly repetitive TEs to which reads cannot be specifically mapped. These points have been clarified in the manuscript text (lines 199-202; 211-213).

In addition, our study investigates TEs at the single (individual) element level, whereas previous studies have investigated families of multiple / many individual TE copies. Therefore, if within a family of 100 TEs, one is highly differentially expressed, previous studies would likely have counted the TE family as differentially regulated, whereas our approach would identify 99/100 as ‘static’ and one as ‘dynamic’.

In looking through the methods, I am wondering if the number of “static” transcripts might be exaggerated by not using an expression cutoff to filter out lowly expressed transcripts that lack power to be called DE and by partially counting ambiguous reads to multiple locations. Although I am not entirely sure from the methods how this analysis was performed since DE TEs were defined using DESeq2, which only allows integer counts, while FeatureCounts is set to output fractions for multi-mapped reads. The methods should also describe how read counts were normalized and what parameters were used to call significantly DE features.

The methods section ‘Analysis of transposable elements’ has been updated with the requested description which clarifies the confusion of ‘not covered’ vs ‘static’ TEs categorization (lines 663-681). The category of TEs with low expression that lack the power to be called DE was categorized as ‘not covered’ TEs, whereas only TEs which passed the ‘Independent Filtering’ of DESeq2 but had an adjustable p-value of >=0.05 (not significant as per our threshold) were categorized as ‘static’ TEs. As noted above, the majority of TEs (>50%) are either ‘not expressed’ or ‘not covered’, and only about one-fifth of the TEs have expression and yet no differential expression across the developmental stages we investigated, and are therefore categorized as static TEs.

Finally, in Figure 2B the authors conclude that dynamic TEs tend to be longer than all TEs. However, it is not clear from this plot how much of this difference in length distribution results from the difference in TE types in the dynamic set. Since LTR Unknown TEs are on average larger than DNA transposons, the increased presence of LTRs alone could explain the pattern. If the authors want to conclude something about TE lengths in the different sets, TE lengths should be compared within each TE type.

At the request of Reviewer #2 we have performed this analysis individually for each TE superfamily. The data demonstrate that our initial “all TEs” observation is true for almost all superfamilies, and we therefore conclude that dynamic TEs are on average longer than all TEs, irrespective of TE type. This analysis and conclusion has been added as Figure S3 (line 218).

***************************************

**Reviewer #3:**

This was a fascinating and clearly written paper. The authors describe an RNA-seq based analysis to confirm that the derepression of transposons in male gametophytes previously reported in arabidopsis appears to be a more general property of angiosperms. They demonstrate the use of a novel screen to quantitatively estimate the effect of KO mutants on pollen fitness in a high throughput fashion and use this screen to qualify fitness effects for 30 genes. There is suggestive but not entirely conclusive evidence that genes which are highly expressed in the pollen vegetative cell are more likely to play rolls in determining pollen fitness. Finally the authors identify and characterize a novel mutant that appears to play a role in controlling the odds of proper double fertilization of both the central cell and egg cell of megagametophytes. The last is an area of growing interest from both a flowering plant evo/devo perspective as well as being an area of economic impact and applied research interest.

As I think is clear from my summary I found this to be a very strong manuscript that will be of interest to a broad audience. I do, however, have a couple of minor concerns/questions:

1) Around like 343 the authors mention that they tested for changes in transmission rate using "h quasi-likelihood tests on generalized linear models with a logit link function for binomial counts." It would be good to include a little discussion of why this test was employed rather than a simple chi-square test.

We added a detailed justification for using our statistical tests to the methods section of the manuscript. (lines 720-729). Most notably, the generalized linear model approach allows the possibility that seeds on the same ear were not completely independent relative to other ears, for example, if there were an effect of temperature at pollination on transmission rate.

2) For Figure 2B and 2D, violin plots may be more informative than simple box plots.

At the request of Reviewer #3 we have changed Figures 2B and 2D to violin plots.

3) Unless I missed it, it doesn't seem like there is any discussion of the fact that genes with significant transmission defects, even only in the male direction, are likely underrepresented in most reverse genetics populations. I would strongly suggest touching on this point in the discussion. If the data are available, it would also be quick and interesting to check whether the genes the authors identify has highly expressed in pollen vegetative cell genes indeed over or under represented in the reverse genetics population employed by the authors (relative to genes highly expressed in seedings or pollen sperm nuclei). But the test is a nice to have, not a must have.

As requested by Reviewer 3, we have added sentences in the Discussion addressing this issue (lines 515-524). In fact, a previous paper from our group (Chettoor et al 2014) extensively assessed the potential for transposable element insertion bias against genes showing enriched expression in gametophytes, and, as this Reviewer suspected, found a significant deficit in insertions in such genes in two large, sequence-indexed *Mutator* populations. We have chosen to not include a similar analysis in this manuscript, as it is already lengthy, and the complexity of a complete analysis (eg, controlling for gene length; exon vs. intron vs. promoter insertions) requires extensive description. However, as we note in the Discussion, the two genes previously published as having severe male transmission defects (and thus examples of the types of mutations most likely to be selected against) are also both highly expressed in the vegetative cells. Our thought is that, although likely biased as predicted by the Reviewer, the *Ds-GFP* population nonetheless serves as an appropriate resource for investigating the potential for relationships between transcriptomic data and phenotypic outcome in the male gametophyte.
